# Plasma Homocysteine as a Biomarker for Cardiovascular Risk Stratification in Children and Adolescents With Type 1 Diabetes Mellitus: A Cross-Sectional Study From Western Algeria

**DOI:** 10.7759/cureus.115169

**Published:** 2026-08-25

**Authors:** Auda Ameur, Charef Latroch, Hiba Naas, Fatima Amraoui, Nacera Aribi, Karim Bouziane Nedjadi

**Affiliations:** 1 Department of Pediatrics B, Amilcar Cabral, University of Oran 1 Ahmed Ben Bella, Oran, DZA; 2 Laboratory of Environment, Natural Plant Substances and Food Technology, Faculty of Natural and Life Sciences, Ahmed Zabana University - Relizane, Relizane, DZA; 3 Faculty of Natural and Life Sciences, Ahmed Zabana University - Relizane, Relizane, DZA

**Keywords:** algeria, cardiovascular disease risk factors, homocysteine, pediatric type 1 diabetes, vascular endothelial dysfunction

## Abstract

Background

Cardiovascular disease (CVD) remains the leading cause of premature morbidity and mortality among individuals living with type 1 diabetes mellitus (T1DM). Although overt cardiovascular events are uncommon during childhood, endothelial dysfunction and subclinical atherosclerosis develop early, making timely cardiovascular risk assessment essential. Plasma homocysteine has emerged as a potential biomarker of vascular injury; however, its clinical value in pediatric T1DM remains controversial, and evidence from African populations is scarce. This study aimed to evaluate plasma homocysteine concentrations in children and adolescents with T1DM and to investigate their relationship with cardiovascular risk profiles and diabetes-related complications.

Methods

A cross-sectional observational study was conducted at the Department of Pediatrics B, in the university hospital center of Oran, Algeria. A total of 50 children and adolescents aged 5-19 years with confirmed T1DM were included. Demographic, anthropometric, clinical, and laboratory data were retrospectively collected from medical records. Plasma total homocysteine (tHcy) concentrations, glycemic control, lipid profile, diabetes duration, insulin therapy, nephropathy, and cardiovascular risk factors were analyzed. Hyperhomocysteinemia prevalence was determined, and its association with cardiovascular risk stratification was assessed using descriptive and inferential statistical analyses.

Results

The study included 27 male (54%) and 23 female patients (46%), and hyperhomocysteinemia was identified in 17 of 50 participants (34%). Plasma tHcy concentrations were significantly higher in participants with HbA1c >7.5% than in those with HbA1c ≤7.5% (18.9 ± 5.8 vs. 11.8 ± 3.9 μmol/L; Student’s t-test, t = 4.44, p < 0.001). Plasma tHcy concentrations were positively correlated with HbA1c (r = 0.61, p < 0.001), diabetes duration (r = 0.49, p = 0.001), LDL cholesterol (r = 0.42, p = 0.004), and triglycerides (r = 0.35, p = 0.015). Participants with diabetic nephropathy had significantly higher plasma tHcy concentrations than those without nephropathy (21.1 ± 5.9 vs. 15.2 ± 5.3 μmol/L; Student’s t-test, t = 2.53, p = 0.015). In exploratory multivariable analysis, poor glycemic control (HbA1c >7.5%; aOR = 3.26, 95% CI: 1.18-9.02; p = 0.021) and diabetes duration ≥5 years (aOR = 2.54, 95% CI: 1.01-6.42; p = 0.047) were independently associated with hyperhomocysteinemia. Plasma tHcy showed good discriminative ability for the study-specific moderate-to-high cardiovascular risk classification (AUC = 0.82, 95% CI: 0.69-0.94; p < 0.001).

Conclusion

Hyperhomocysteinemia was prevalent among children and adolescents with T1DM in our cohort and was associated with an unfavorable cardiovascular risk-factor profile. These findings suggest that plasma homocysteine may represent a potential investigational adjunctive marker of cardiovascular risk-factor burden in pediatric T1DM. Prospective multicenter studies are warranted to validate these associations and determine their incremental clinical value beyond established cardiovascular risk factors.

## Introduction

Type 1 diabetes mellitus (T1DM) is a lifelong autoimmune disorder and one of the leading endocrine diseases affecting children and adolescents worldwide [1-4]. Despite major advances in insulin therapy, continuous glucose monitoring, and automated insulin delivery, children with T1DM remain vulnerable to long-term vascular complications whose underlying pathological processes may begin years before clinical manifestations become evident [5-8]. Although overt cardiovascular events are uncommon during childhood, vascular injury may begin early, emphasizing the importance of cardiovascular risk assessment during the asymptomatic stage [9].

Cardiovascular complications in T1DM result from a complex interaction between persistent hyperglycemia, oxidative stress, chronic inflammation, and endothelial dysfunction [10,11]. These processes may progressively alter vascular structure and function, with arterial stiffening and increased carotid intima-media thickness reported before clinically overt cardiovascular disease develops [12-14]. Cardiovascular risk assessment in pediatric T1DM therefore relies primarily on established clinical and metabolic factors, including glycated hemoglobin (HbA1c), diabetes duration, obesity, hypertension, dyslipidemia, albuminuria, tobacco smoke exposure, and family history of premature cardiovascular disease [3,15]. However, these conventional factors may not fully account for the heterogeneity of cardiovascular risk-factor burden among patients with T1DM [16,17], prompting interest in additional circulating biomarkers that may provide complementary information [18].

Homocysteine is a sulfur-containing amino acid formed during methionine metabolism. Its plasma concentration is regulated through remethylation and transsulfuration pathways involving folate, vitamin B12, vitamin B6, and several enzymes involved in one-carbon metabolism [19]. Elevated homocysteine concentrations have been associated with oxidative stress, endothelial dysfunction, impaired nitric oxide synthesis, and increased thrombotic activity [10,11,20,21]. These biological effects provide a rationale for investigating the relationship between plasma homocysteine and diabetes-related vascular abnormalities.

However, the role of plasma homocysteine as a cardiovascular biomarker in pediatric T1DM remains uncertain. Although hyperhomocysteinemia has been extensively investigated as a cardiovascular risk marker in adults with T1DM, its clinical significance remains debated because interventions aimed at lowering homocysteine concentrations have not consistently reduced cardiovascular events [20,21]. Evidence in children and adolescents is substantially more limited and remains inconsistent. Several studies have reported associations between elevated homocysteine concentrations and poor glycemic control, diabetic nephropathy, subclinical vascular abnormalities, and diabetes-related complications [22,23]. Conversely, Wiltshire et al. reported normal or lower homocysteine concentrations in well-controlled pediatric patients [24]. Differences in age, nutritional status, renal function, metabolic control, insulin therapy, and genetic factors may contribute to these conflicting findings. Importantly, biological associations between elevated homocysteine and cardiovascular risk factors do not necessarily imply independent predictive or prognostic utility. Plasma homocysteine should therefore currently be regarded as a risk-associated, investigational biomarker rather than an established predictor of cardiovascular outcomes in pediatric T1DM. Collectively, the available evidence indicates that cardiovascular risk in T1DM is multifactorial and that homocysteine should be interpreted within a broader metabolic and clinical context.

## Materials and methods

Study design

This cross-sectional observational study was conducted to investigate the association between plasma total homocysteine (tHcy) concentrations and cardiovascular risk among children and adolescents with T1DM. Plasma tHcy was selected as the primary biomarker because accumulating evidence supports its role as an early indicator of endothelial dysfunction and subclinical atherosclerosis in pediatric and adolescent populations [23,24]. The study was designed and reported in accordance with the Strengthening the Reporting of Observational Studies in Epidemiology (STROBE) guidelines for observational studies [25].

Study setting

The study was carried out at the Pediatric Diabetes Unit, Department of Pediatrics B, University Hospital of Oran, Algeria, a tertiary referral center specializing in the diagnosis, management, and long-term follow-up of children and adolescents with diabetes mellitus. Data were collected over a three-month period through a systematic review of electronic and paper-based medical records, together with standardized clinical follow-up forms routinely completed during outpatient diabetes consultations.

Study population

Eligible participants were children and adolescents diagnosed with T1DM according to the diagnostic criteria established by the American Diabetes Association (ADA). Consecutive patients attending the Pediatric Diabetes Unit during the study period were screened for eligibility. A total of 50 patients meeting all predefined eligibility criteria were included in the final analysis [1].

The inclusion and exclusion criteria used for participant selection are summarized in Table 1, which illustrates the study population selection process.

**Table 1:** Inclusion and Exclusion Criteria Applied for Participant Selection. Inclusion and exclusion criteria used to identify eligible children and adolescents with type 1 diabetes mellitus enrolled in this cross-sectional study. T1DM, type 1 diabetes mellitus; T2DM, type 2 diabetes mellitus.

Inclusion Criteria	Exclusion Criteria
Children and adolescents aged 5–19 years	T2DM, monogenic diabetes, or secondary diabetes
Confirmed diagnosis of T1DM	Chronic inflammatory or autoimmune diseases unrelated to T1DM
Regular follow-up at the Pediatric Diabetes Unit, University Hospital of Oran	Chronic liver disease
Availability of complete clinical and laboratory data, including plasma homocysteine measurement	Severe renal impairment unrelated to diabetic nephropathy
Informed consent obtained from parents or legal guardians	Vitamin B12 or folate supplementation within the previous 3 months
	Incomplete clinical or laboratory records

Data collection

Clinical, demographic, anthropometric, and laboratory data were retrospectively extracted from the medical records of all eligible participants using a standardized data collection form. Demographic variables included age, sex, region of residence, and educational level. Anthropometric assessment comprised body weight, height, and body mass index (BMI), which was calculated as weight in kilograms divided by the square of height in meters (kg/m²). BMI categories were interpreted according to the World Health Organization (WHO) 2007 growth reference for children and adolescents aged 5-19 years [26].

Clinical characteristics related to T1DM included age at diagnosis, duration of diabetes, insulin regimen, total daily insulin dose, level of physical activity, family history of diabetes mellitus, and family history of premature cardiovascular disease.

Laboratory and Clinical Assessment

Laboratory data collected from routine clinical evaluations included HbA1c, plasma tHcy, lipid profile (total cholesterol, low-density lipoprotein cholesterol, high-density lipoprotein cholesterol, and triglycerides), renal function parameters, and the presence or absence of diabetic nephropathy. Plasma tHcy was measured as part of routine biochemical assessment performed in the hospital clinical laboratory using absorption spectrophotometry on a Roche cobas c501 analyzer (Roche Diagnostics). Hyperhomocysteinemia was defined as a plasma tHcy concentration >15 μmol/L, based on the upper reference limit used by the performing laboratory. This laboratory-defined threshold was applied uniformly to all participants. Detailed information regarding fasting status and preanalytical sample handling was not consistently available in the retrospective medical records.

Glycemic control was assessed according to the current International Society for Pediatric and Adolescent Diabetes (ISPAD) recommendations. Poor glycemic control was defined as an HbA1c value above the recommended target for children and adolescents with T1DM [2-4].

Renal involvement was assessed from routinely available clinical and laboratory data, including renal function parameters and the documented presence or absence of diabetic nephropathy. Because detailed renal indices were not systematically available for all participants, renal function was considered as a potential source of residual confounding in the interpretation of plasma tHcy concentrations.

Cardiovascular risk was assessed using a study-specific composite assessment based exclusively on seven established clinical and metabolic cardiovascular risk factors relevant to pediatric T1DM: poor glycemic control (HbA1c >7.5%), diabetes duration ≥5 years, overweight or obesity, dyslipidemia, hypertension, diabetic nephropathy, and a family history of premature cardiovascular disease. One point was assigned for each risk factor present, resulting in a cumulative risk-factor count ranging from 0 to 7. Participants with 0-1 risk factors were classified as low risk, those with 2-3 risk factors as moderate risk, and those with ≥4 risk factors as high risk. This composite assessment was developed for exploratory stratification within the present study and does not represent an externally validated cardiovascular risk prediction score. Importantly, plasma tHcy was evaluated separately as the biomarker of interest and was not included in the construction of the composite cardiovascular risk categories, thereby ensuring that the cardiovascular risk classification used as the reference outcome for ROC analysis was determined independently of plasma tHcy concentration.

The primary outcome of the study was the prevalence of hyperhomocysteinemia among children and adolescents with T1DM. Secondary outcomes included the associations between plasma tHcy concentrations and glycemic control, diabetes duration, lipid abnormalities, diabetic nephropathy, and the study-specific composite cardiovascular risk categories.

Statistical analysis

Statistical analyses were performed using IBM SPSS Statistics for Windows, Version 30.0 (IBM Corp., Armonk, NY, USA). Continuous variables were expressed as mean ± standard deviation (SD) or median (interquartile range [IQR]), according to their distribution, while categorical variables were presented as frequencies and percentages. Between-group comparisons were performed using Student’s t-test or the Mann-Whitney U test for continuous variables and the chi-square test or Fisher’s exact test for categorical variables, as appropriate. Correlations between plasma tHcy concentrations and quantitative variables were assessed using Pearson’s or Spearman’s correlation coefficients, according to data distribution. Variables significantly associated with hyperhomocysteinemia in univariate analyses were subsequently entered into a multivariable logistic regression model to identify independent predictors. Receiver operating characteristic (ROC) curve analysis was performed to evaluate the discriminative ability of plasma tHcy to identify participants with increased cardiovascular risk based on the study-specific composite cardiovascular risk classification. Importantly, plasma tHcy was not included in the construction of the composite risk categories; therefore, the cardiovascular risk classification used as the reference outcome for ROC analysis was determined independently of plasma tHcy concentration. The area under the ROC curve (AUC) and corresponding 95% confidence interval (CI) were calculated. A two-sided p-value <0.05 was considered statistically significant.

Ethical considerations

The study was conducted in accordance with the ethical principles of the Declaration of Helsinki. Because this investigation was based on anonymized retrospective clinical data routinely collected during patient follow-up, the confidentiality and privacy of patient information were strictly maintained throughout the study.

The study protocol was reviewed and approved by the Ethics Committee of the Faculty of Medicine, University of Oran 1 Ahmed Ben Bella, Oran, Algeria, prior to data collection. As the study involved retrospective analysis of fully anonymized data, the requirement for informed consent was waived by the Ethics Committee.

## Results

Baseline characteristics

A total of 50 children and adolescents with T1DM were included in the study. The mean age was 12.9 ± 3.6 years (range, 5-19 years). Twenty-seven participants (54.0%) were male, and twenty-three (46.0%) were female, corresponding to a male-to-female ratio of 1.17.

The median duration of diabetes was 5.2 years (IQR, 2.4-8.1 years), ranging from a few months to 16 years. Nineteen patients (38.0%) had diabetes for 1-5 years, 17 (34.0%) for 6-10 years, 9 (18.0%) for more than 10 years, and 5 (10.0%) for less than one year. Nearly three-quarters of the participants (72.0%) were residents of Oran Province.

The mean body mass index (BMI) was 20.8 ± 3.7 kg/m². According to the World Health Organization (WHO) growth references, 32 participants (64.0%) had a normal BMI, 10 (20.0%) were overweight, 4 (8.0%) were obese, and 4 (8.0%) were underweight.

Glycemic control and cardiovascular risk profile

The mean HbA1c level was 9.46 ± 2.01% (95% confidence interval [CI], 8.90-10.02%). Overall, only 16 participants (32.0%) achieved the recommended glycemic target (HbA1c ≤7.5%), whereas 34 (68.0%) had HbA1c values above 7.5%, indicating suboptimal metabolic control.

A family history of premature cardiovascular disease was reported in 18 patients (36.0%). Diabetic nephropathy was present in 6 participants (12.0%), while the remaining 44 (88.0%) showed no clinical evidence of renal involvement.

Plasma homocysteine concentrations

**Figure 1:**
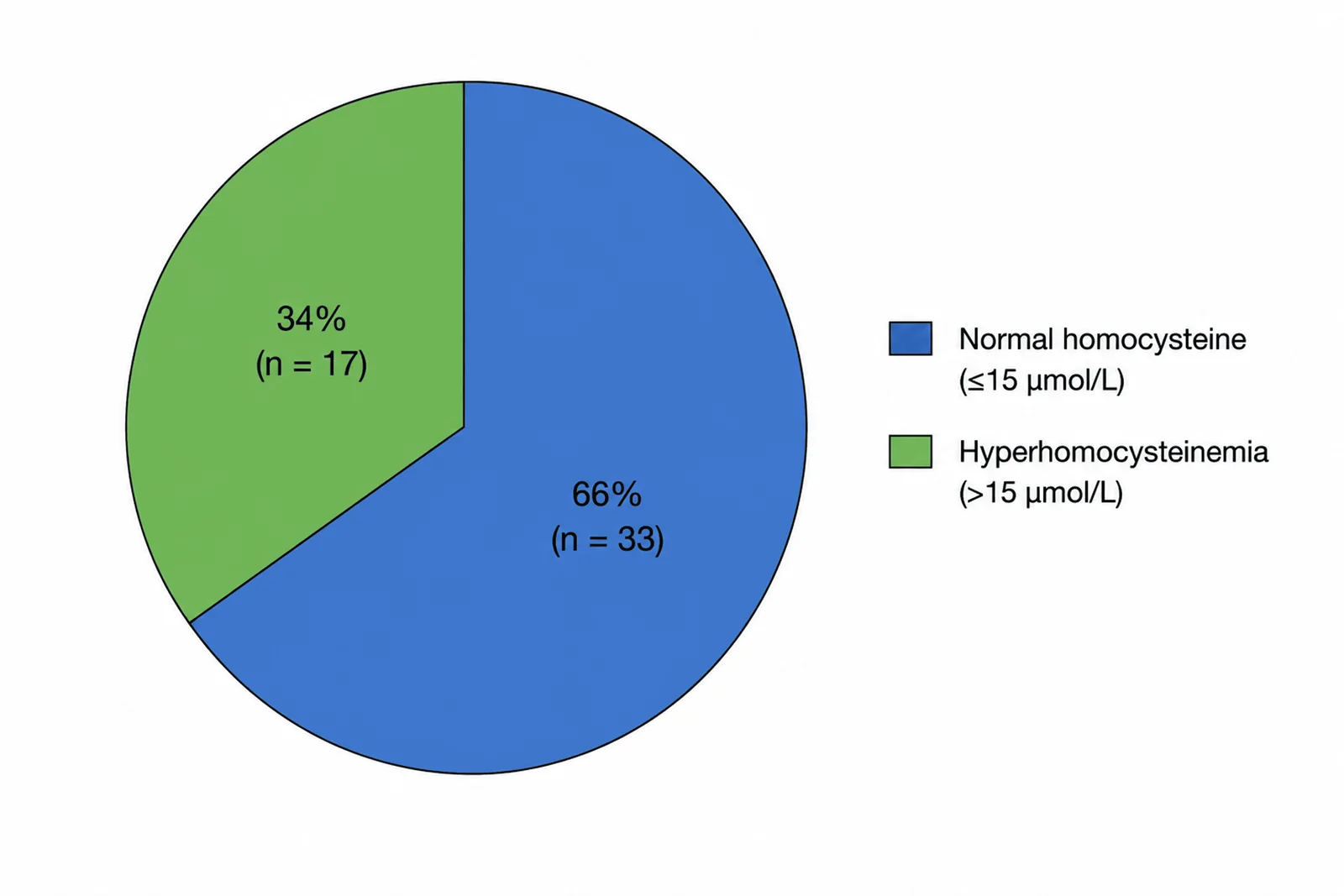
Distribution of plasma homocysteine status among study participants. Hyperhomocysteinemia was observed in 17 (34%) participants, while 33 (66%) had normal plasma homocysteine concentrations. Image Credits: Auda Ameur. Figure created by the author using IBM SPSS Statistics version 30.0 and Microsoft PowerPoint.

Plasma homocysteine concentrations ranged from 6 to 33 μmol/L, with a mean concentration of 16.8 ± 6.4 μmol/L. Based on the predefined laboratory reference values, 17 patients (34.0%) had hyperhomocysteinemia, whereas 33 patients (66.0%) had homocysteine concentrations within the normal range. Patients with hyperhomocysteinemia had a significantly longer duration of diabetes than those with normal homocysteine levels (6.8 ± 3.4 vs. 3.9 ± 2.5 years, p = 0.003; independent-samples Student’s t-test). The distribution of plasma homocysteine status among the study participants is shown in Figure 1.

Association between plasma homocysteine and glycemic control

**Figure 2:**
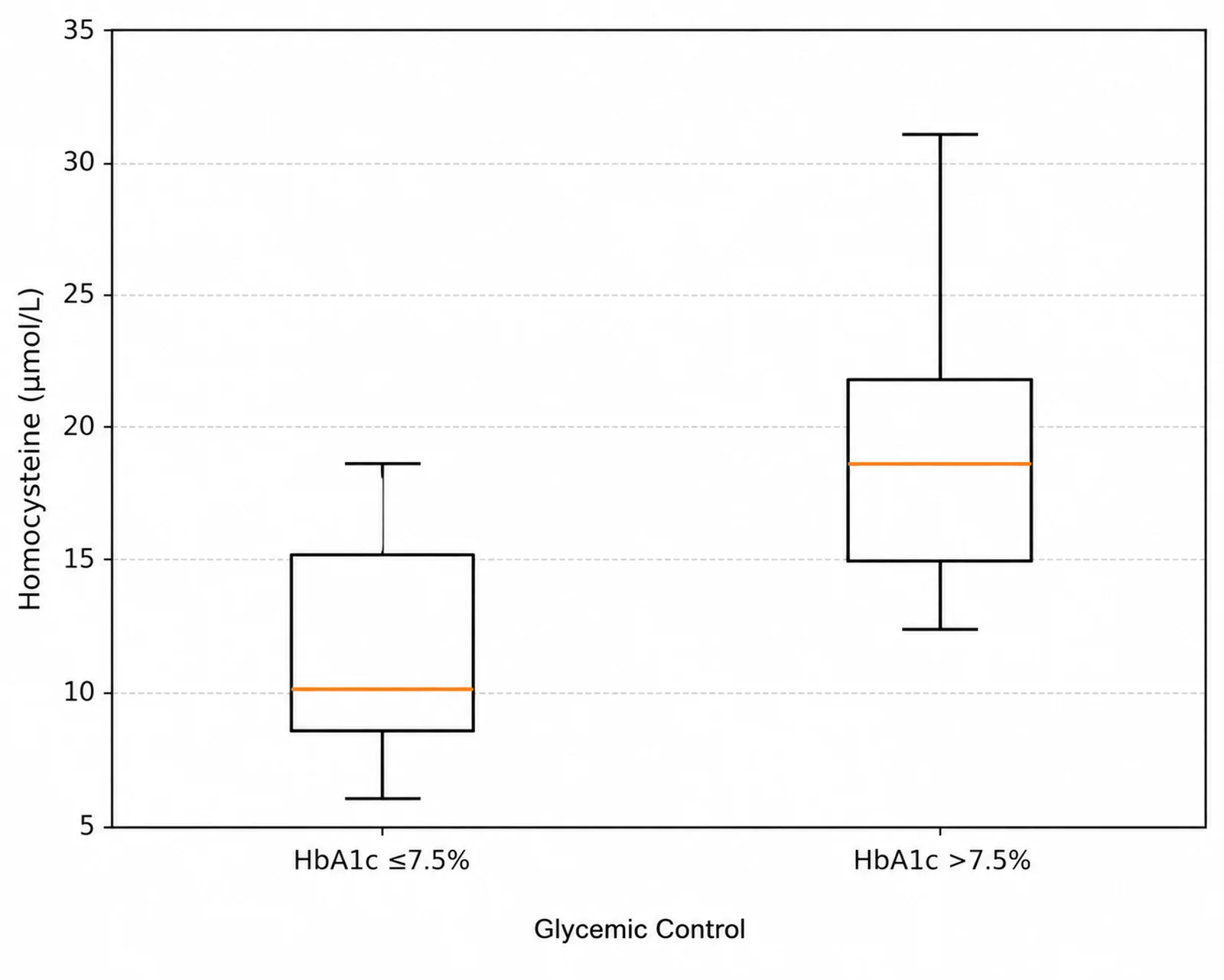
Plasma homocysteine concentrations according to glycemic control. Boxplots compare plasma homocysteine concentrations between participants with good glycemic control (HbA1c ≤7.5%) and poor glycemic control (HbA1c >7.5%). The central line represents the median, the box indicates the interquartile range (IQR), and the whiskers represent the minimum and maximum observed values. Image Credit: Auda Ameur. **Figure prepared using IBM SPSS Statistics for Windows, Version 30.0 (IBM Corp., Armonk, NY, USA).

Plasma homocysteine concentrations were significantly associated with glycemic control. Participants with poor metabolic control (HbA1c >7.5%) exhibited substantially higher homocysteine concentrations than those achieving the recommended glycemic target (Figure 2). The mean homocysteine concentration was 11.8 ± 3.9 μmol/L in patients with HbA1c ≤7.5%, compared with 18.9 ± 5.8 μmol/L in those with HbA1c >7.5%. This difference was statistically significant (Student's t-test, t = 4.44, p < 0.001).

Correlation between plasma homocysteine and clinical variables

Pearson correlation analysis demonstrated a significant positive correlation between plasma homocysteine concentration and HbA1c (r = 0.61, p < 0.001). A moderate positive correlation was also observed between homocysteine concentration and diabetes duration (r = 0.49, p = 0.001).

**Figure 3:**
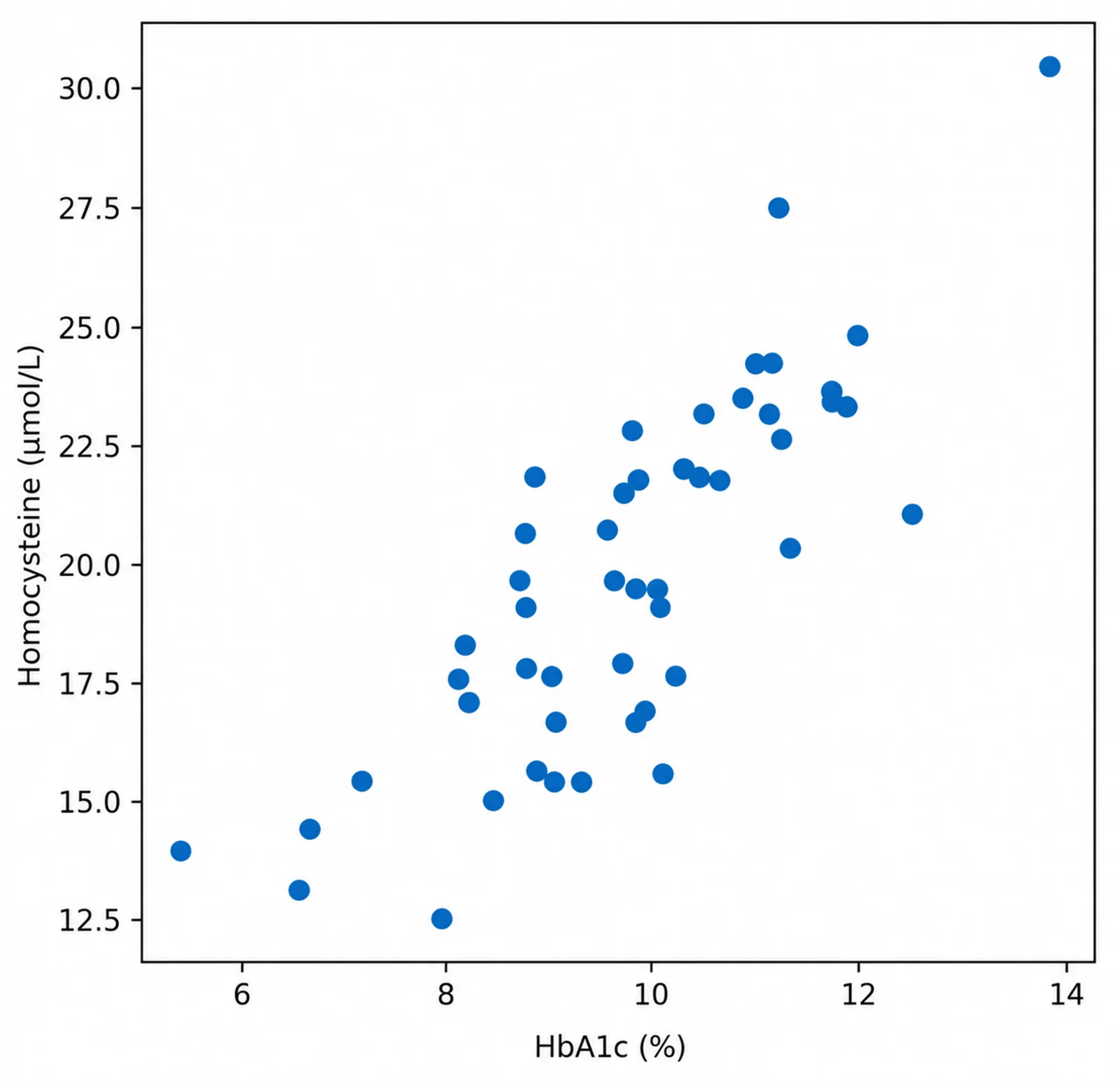
Scatter plot showing the relationship between HbA1c and plasma homocysteine concentrations. Each point represents one participant. A positive linear relationship is observed, indicating increasing plasma homocysteine concentrations with worsening glycemic control.

**Figure 4:**
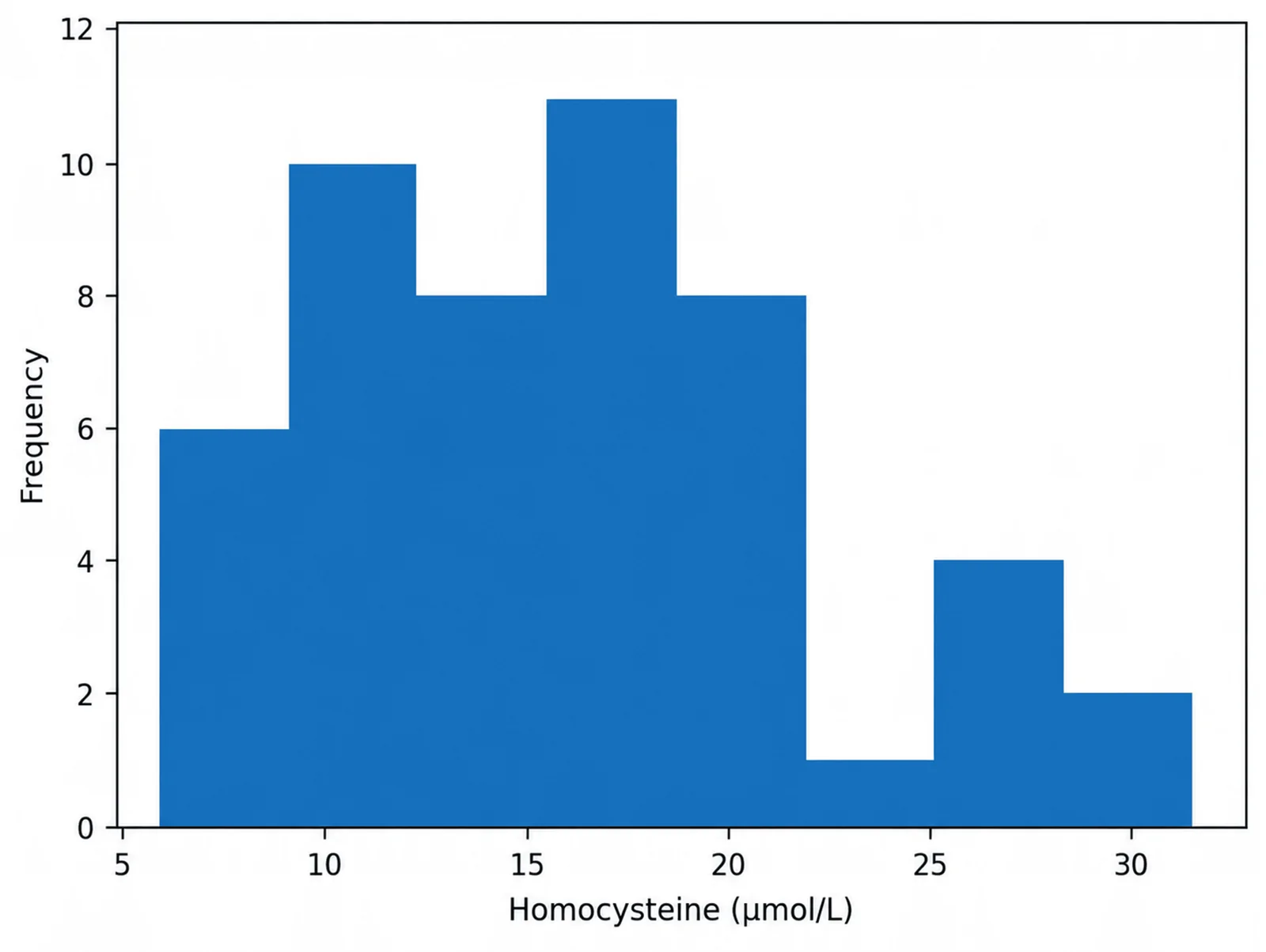
Distribution of plasma homocysteine concentrations. Histogram illustrating the frequency distribution of plasma homocysteine concentrations among the 50 participants.

Homocysteine levels were positively correlated with LDL cholesterol (r = 0.42, p = 0.004) and triglyceride concentrations (r = 0.35, p = 0.015). No significant association was found between homocysteine concentration and BMI (r = 0.19, p = 0.18). The relationship between HbA1c and plasma homocysteine concentrations is illustrated in Figure 3. The overall distribution of plasma homocysteine concentrations is shown in Figure 4.

Plasma homocysteine according to diabetic nephropathy

Six participants (6/50, 12.0%) had diabetic nephropathy. Patients with diabetic nephropathy exhibited significantly higher plasma homocysteine concentrations than those without renal involvement (21.1 ± 5.9 vs. 15.2 ± 5.3 μmol/L, Student's t-test, t = 2.53, p = 0.015).

Composite cardiovascular risk assessment

A study-specific composite cardiovascular risk assessment was performed among the 17 patients (34.0%) with hyperhomocysteinemia using seven established cardiovascular risk factors: poor glycemic control (HbA1c >7.5%), diabetes duration ≥5 years, overweight or obesity, dyslipidemia, hypertension, diabetic nephropathy, and a family history of premature cardiovascular disease. One point was assigned for each risk factor present, resulting in a cumulative risk-factor count ranging from 0 to 7.

Among the 17 patients with hyperhomocysteinemia, poor glycemic control was the most prevalent cardiovascular risk factor, affecting 16 patients (94.1%), followed by diabetes duration ≥5 years in 14 patients (82.4%), overweight or obesity in 13 patients (76.5%), and a family history of premature cardiovascular disease in 13 patients (76.5%). Diabetic nephropathy was present in 5 patients (29.4%), whereas dyslipidemia and hypertension were each identified in 2 patients (11.8%).

The median cumulative risk-factor count was 4 (IQR, 3-5). According to the predefined study-specific classification, 2 patients (11.8%) were categorized as low risk, 7 patients (41.2%) as moderate risk, and 8 patients (47.1%) as high risk.

Overall, 15 of the 17 patients (88.2%) with hyperhomocysteinemia were classified within the moderate- or high-risk categories. The distribution of hyperhomocysteinemia differed significantly across the study-specific cardiovascular risk categories (χ² = 10.8, p = 0.005), supporting an association between elevated tHcy and a greater cumulative burden of established cardiovascular risk factors.

Discriminative performance of plasma homocysteine

**Figure 5:**
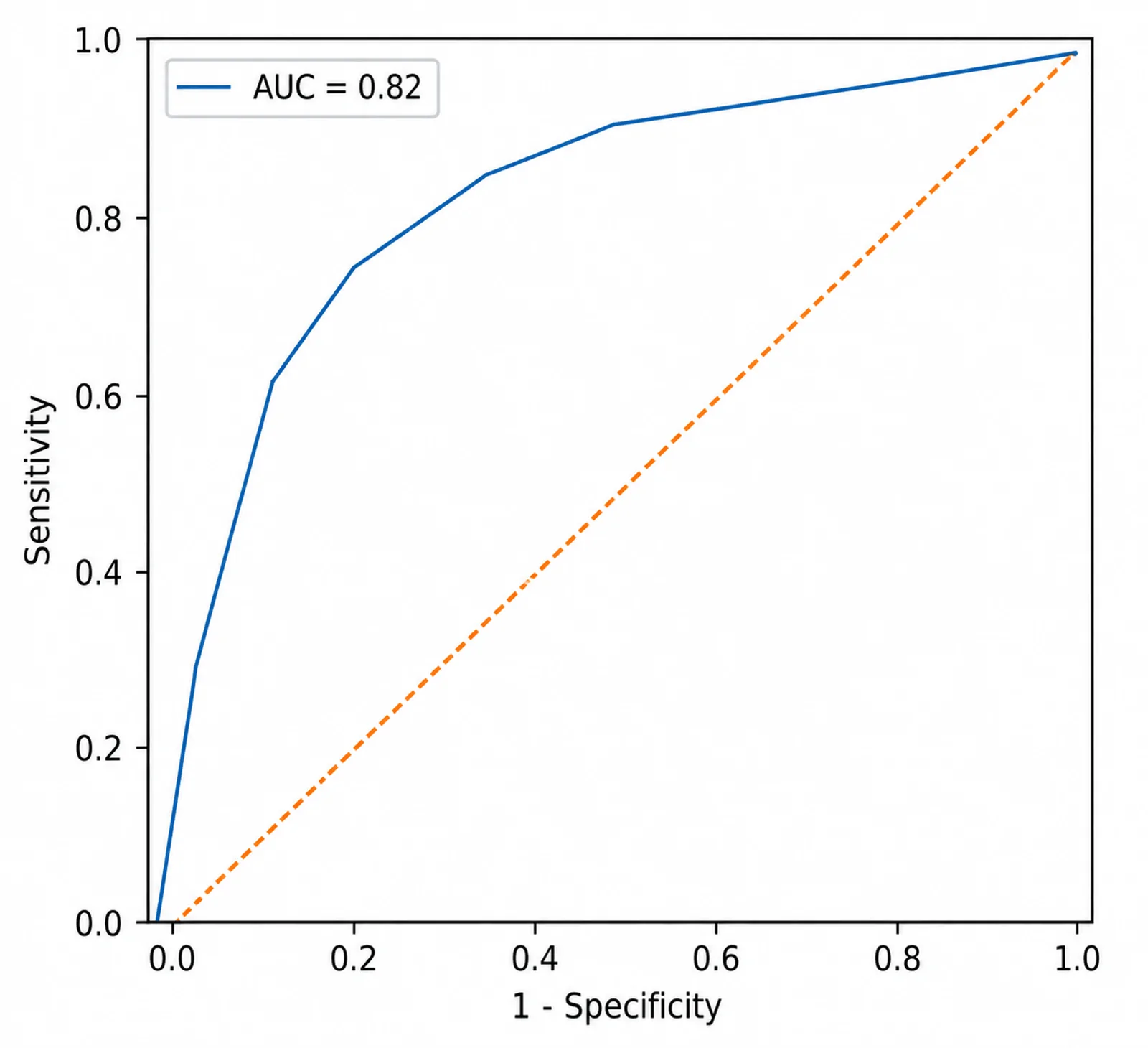
ROC curve of plasma tHcy for cardiovascular risk prediction. ROC curve of plasma tHcy for discrimination of the study-specific moderate-to-high cardiovascular risk-factor category. ROC: Receiver operating characteristic; tHcy: total homocysteine; AUC: area under the receiver operating characteristic curve.

ROC curve analysis was performed to evaluate the ability of plasma tHcy to discriminate participants classified as having moderate-to-high cardiovascular risk according to the study-specific composite cardiovascular risk assessment (Figure 5). Importantly, the composite risk classification used as the reference outcome was determined independently of plasma tHcy concentration.

Plasma tHcy demonstrated good discriminative performance, with an area under the ROC curve (AUC) of 0.82 (95% confidence interval [CI]: 0.69-0.94; p < 0.001). The optimal exploratory cutoff was 16.5 μmol/L, with a sensitivity of 82% and specificity of 76%. Given the limited sample size, these classification performance estimates and the identified cutoff should be interpreted cautiously and require validation in larger independent cohorts.

Overall, plasma tHcy demonstrated discriminative ability for distinguishing participants with a greater burden of established cardiovascular risk factors within this study-specific classification framework. However, these ROC findings are exploratory and should not be interpreted as evidence of predictive or prognostic performance.

Correlation analyses

Pearson correlation analysis revealed significant positive associations between plasma tHcy concentrations and several established cardiovascular risk markers. The strongest correlation was observed between tHcy and HbA1c (r = 0.61, p < 0.001), indicating that higher tHcy concentrations were associated with poorer glycemic control. A moderate positive correlation was also identified between tHcy and diabetes duration (r = 0.49, p = 0.001), indicating higher tHcy concentrations with longer diabetes duration.

Significant positive correlations were also observed between tHcy and LDL cholesterol (r = 0.42, p = 0.004) and triglycerides (r = 0.35, p = 0.015). In contrast, no statistically significant correlation was observed between tHcy and body mass index (BMI) (r = 0.19, p = 0.18).

**Figure 6:**
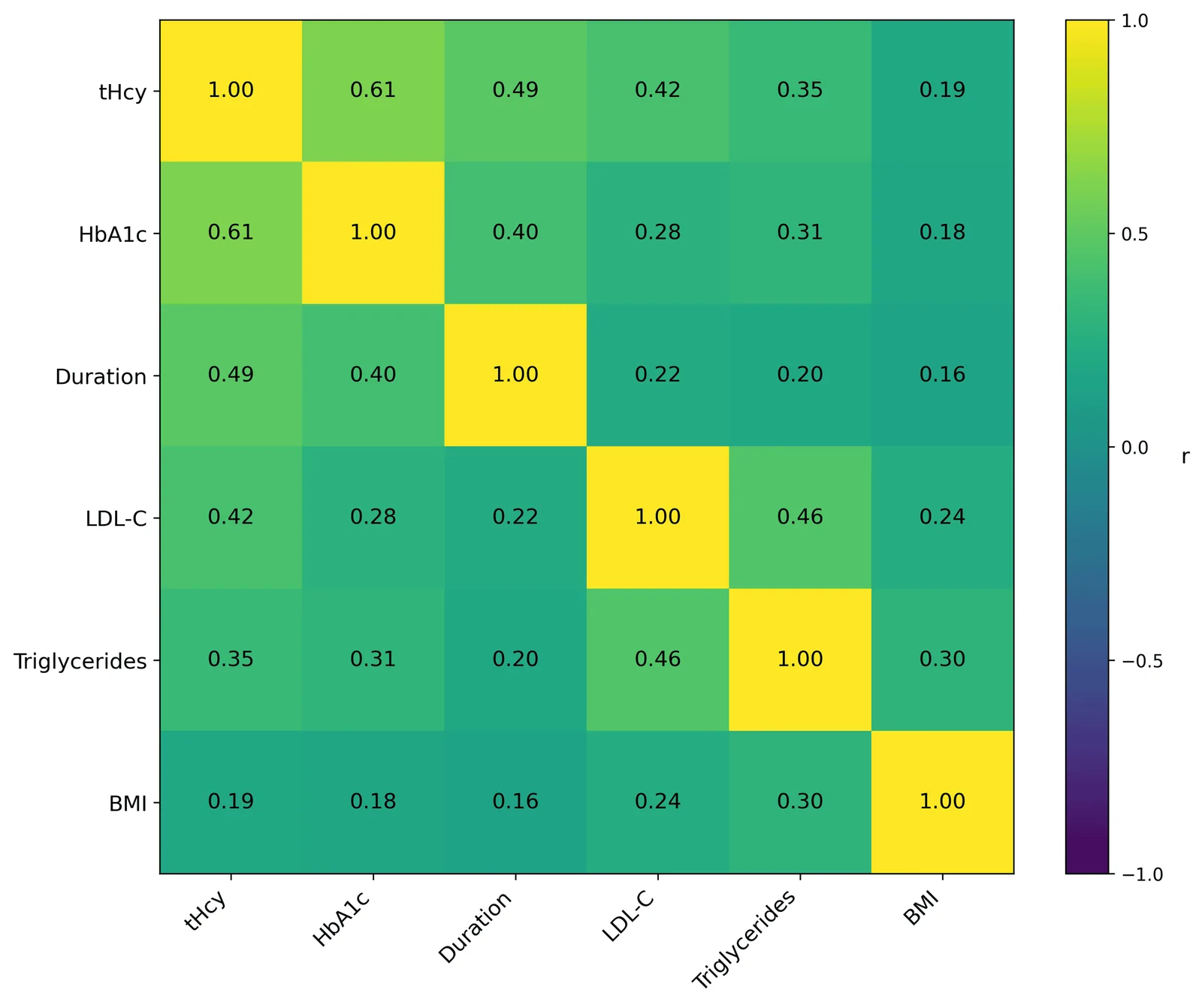
Correlation matrix of plasma homocysteine and cardiovascular risk variables. Heatmap illustrating Pearson correlation coefficients between plasma homocysteine, HbA1c, diabetes duration, LDL cholesterol, and BMI. Stronger positive correlations are represented by warmer colors.

Overall, these findings indicate that higher plasma tHcy concentrations were associated with poorer glycemic control, longer diabetes duration, and a less favorable lipid profile, whereas no significant association was observed with BMI. The correlation matrix summarizing these relationships is shown in Figure 6.

Association with diabetic nephropathy

Diabetic nephropathy was identified in six participants (12.0%). Participants with nephropathy exhibited significantly higher plasma tHcy concentrations than those without nephropathy. The mean plasma tHcy concentration was 21.1 ± 5.9 μmol/L in participants with nephropathy compared with 15.2 ± 5.3 μmol/L in those without nephropathy. This difference was statistically significant (Student’s t-test, p = 0.015), indicating an association between higher plasma tHcy concentrations and the presence of diabetic nephropathy.

Multivariable logistic regression analysis

A multivariable logistic regression analysis was performed to explore factors independently associated with hyperhomocysteinemia. After adjustment for potential confounding variables, poor glycemic control (HbA1c >7.5%) showed the strongest independent association with hyperhomocysteinemia (adjusted odds ratio [aOR] = 3.26, 95% CI: 1.18-9.02, p = 0.021). Diabetes duration ≥5 years was also independently associated with hyperhomocysteinemia (aOR = 2.54, 95% CI: 1.01-6.42, p = 0.047).

Although overweight or obesity was associated with increased odds of hyperhomocysteinemia (aOR = 2.11, 95% CI: 0.81-5.48), this association did not reach statistical significance (p = 0.128). Similarly, sex was not independently associated with hyperhomocysteinemia (aOR = 1.18, 95% CI: 0.44-3.12, p = 0.74). Overall, poor glycemic control and longer diabetes duration remained independently associated with hyperhomocysteinemia after multivariable adjustment, consistent with the greater burden of cardiovascular risk factors observed among participants with elevated tHcy. Given the relatively small sample size and the limited number of participants with hyperhomocysteinemia, however, the multivariable regression findings should be interpreted as exploratory and require confirmation in larger cohorts.

## Discussion

The present study provides new evidence regarding the potential role of plasma homocysteine as a marker of cardiovascular risk in children and adolescents with T1DM from Western Algeria. Hyperhomocysteinemia was detected in 17 of 50 participants (34%) and was consistently associated with poor glycemic control, longer disease duration, diabetic nephropathy, and a greater burden of cardiovascular risk factors. In addition, plasma homocysteine demonstrated good discriminatory performance for identifying patients at moderate-to-high cardiovascular risk, suggesting that it may complement conventional clinical and biochemical markers used in pediatric diabetes care [18-21].

To facilitate interpretation of our findings, Table 2 summarizes the principal clinical studies and international recommendations addressing cardiovascular risk and homocysteine in T1DM. Although differences in study populations, age groups, and methodological approaches limit direct comparisons, previous investigations consistently indicate that vascular injury begins early in the natural history of T1DM and progresses under the combined influence of chronic hyperglycemia, oxidative stress, endothelial dysfunction, and cumulative metabolic exposure [4-8,16,17,27-29]. Within this context, our study expands the available evidence by providing one of the first pediatric datasets from North Africa evaluating plasma homocysteine as a potential adjunctive biomarker for cardiovascular risk stratification.

**Table 2:** Overview of Key Clinical Studies and International Guidelines Relevant to Plasma Homocysteine and Cardiovascular Risk in T1DM. T1DM, type 1 diabetes mellitus; DCCT, Diabetes Control and Complications Trial; EDIC, Epidemiology of Diabetes Interventions and Complications; EURODIAB, European Diabetes Complications Study; SEARCH, SEARCH for Diabetes in Youth Study; ISPAD, International Society for Pediatric and Adolescent Diabetes; ADA, American Diabetes Association; HbA1c, glycated hemoglobin; T2DM, type 2 diabetes mellitus.

Study (Year)	Population	Sample Size	Main Objective	Key Findings	Comparison With the Present Study
Ameur et al. (2026) (Present study)	Children and adolescents with T1DM	50	Evaluate plasma homocysteine as a biomarker for cardiovascular risk stratification	Hyperhomocysteinemia was identified in 34% (17/50) of patients and was significantly associated with poor glycemic control, longer diabetes duration, diabetic nephropathy, and higher cardiovascular risk.	First pediatric study from North Africa investigating plasma homocysteine as a biomarker for cardiovascular risk stratification in T1DM.
DCCT (1993) [29]	Adolescents and adults with T1DM	1,441	Evaluate the effect of intensive glycemic control on diabetic complications	Intensive insulin therapy reduced the risk of microvascular complications by 35–76%.	Supports our finding that poor glycemic control is strongly associated with elevated homocysteine and increased cardiovascular risk.
Vurallı et al. (2024) [21]	Adolescents with T1DM	80	Evaluate cardiovascular risk factors in adolescents with T1DM	Poor glycemic control, dyslipidemia, and excess body weight were identified as major cardiovascular risk factors.	Consistent with the association between poor metabolic control and elevated homocysteinemia observed in our study.
ISPAD Clinical Practice Consensus Guidelines (2022) [4]	International pediatric recommendations	Consensus	Prevention and screening of diabetes-related vascular complications	Recommend early assessment of HbA1c, blood pressure, lipid profile, renal function, and healthy lifestyle behaviors.	Our findings suggest that plasma homocysteine may complement current cardiovascular risk assessment in selected high-risk pediatric patients.
ADA Standards of Care (2026) [1]	International recommendations	Consensus	Cardiovascular risk assessment and management in children and adolescents with T1DM	Emphasize optimal glycemic control and management of conventional cardiovascular risk factors; routine homocysteine measurement is not currently recommended.	Our findings suggest that homocysteine may provide additional prognostic information in patients with poor metabolic control or multiple cardiovascular risk factors.
Wiltshire et al. (2001) [24]	Children and adolescents with T1DM	58	Evaluate plasma homocysteine concentrations in pediatric T1DM	Plasma homocysteine concentrations were generally within the normal range in well-controlled children without diabetic nephropathy.	Differences may be explained by better metabolic control, shorter disease duration, and the absence of chronic diabetic complications compared with our cohort.
Rawshani et al. (2018) [5]	Swedish National Diabetes Register	>27,000	Evaluate long-term cardiovascular outcomes according to age at T1DM onset	Childhood-onset T1DM was associated with markedly increased lifetime cardiovascular morbidity and mortality.	Reinforces the need for early biomarkers capable of identifying children at increased cardiovascular risk.
Vergès (2020) [27]	Narrative review	—	Review the epidemiology and mechanisms of cardiovascular disease in T1DM	Chronic hyperglycemia, oxidative stress, inflammation, endothelial dysfunction, and metabolic memory contribute to accelerated atherosclerosis.	Provides biological support for the association between hyperhomocysteinemia and cardiovascular risk observed in our study.
Recent Systematic Reviews and Meta-analyses (2022–2025) [8,18–20]	Individuals with T1DM and T2DM	>10,000 participants	Evaluate the association between plasma homocysteine and cardiovascular disease	Elevated plasma homocysteine was consistently associated with endothelial dysfunction, atherosclerosis, and increased cardiovascular risk.	Consistent with our findings supporting plasma homocysteine as a promising adjunctive biomarker for cardiovascular risk stratification.

The prevalence of hyperhomocysteinemia observed in our cohort is consistent with reports indicating that disturbances in homocysteine metabolism become increasingly apparent with longer disease duration and worsening metabolic control [22]. By contrast, Wiltshire et al. [24] reported predominantly normal homocysteine concentrations in children with well-controlled T1DM and preserved renal function. These discrepancies are likely explained by differences in metabolic control, diabetes duration, and the presence of early diabetes-related complications between study populations. Collectively, these observations support the hypothesis that plasma homocysteine reflects cumulative metabolic stress rather than representing an isolated abnormality, reinforcing its potential value as an indicator of early vascular impairment in pediatric T1DM [23].

Among all the variables examined, glycemic control emerged as the factor most strongly associated with plasma homocysteine concentrations. Children with HbA1c values above the recommended target exhibited significantly higher homocysteine levels, and poor metabolic control remained the principal independent predictor of hyperhomocysteinemia after multivariable adjustment. These findings are biologically plausible because sustained hyperglycemia promotes oxidative stress, chronic inflammation, advanced glycation end-product formation, and endothelial dysfunction, all of which may interfere with homocysteine metabolism and accelerate vascular injury [10,11,18,20,21]. Rather than acting as an isolated pathogenic factor, elevated plasma homocysteine may therefore reflect the cumulative metabolic burden associated with persistent hyperglycemia.

Our observations are consistent with previous evidence demonstrating the long-term vascular consequences of glycemic exposure and the burden of cardiovascular and diabetes-related complications in T1DM [27]. Dabelea et al. demonstrated a substantial burden of diabetes-related complications among individuals diagnosed with diabetes during childhood and adolescence [28]. Consistent with these observations, the Diabetes Control and Complications Trial (DCCT) and its Epidemiology of Diabetes Interventions and Complications (EDIC) follow-up established that intensive glycemic control substantially reduces the incidence of microvascular complications and confers durable cardiovascular protection through the phenomenon of metabolic memory [17,29]. Although these landmark studies did not specifically investigate plasma homocysteine, they clearly demonstrated that prolonged exposure to poor glycemic control has lasting adverse effects on the vascular system. The strong association observed between HbA1c and plasma homocysteine in our cohort supports the hypothesis that homocysteine may serve as a biochemical indicator of cumulative vascular stress rather than simply reflecting short-term metabolic status.

Diabetes duration also showed an independent relationship with hyperhomocysteinemia, suggesting that prolonged exposure to the diabetic milieu may contribute to progressive metabolic and vascular alterations from childhood onward [27-29]. Likewise, participants with diabetic nephropathy had significantly higher plasma homocysteine concentrations than those without renal involvement, highlighting the close interplay between renal function and homocysteine metabolism. Reduced renal clearance, together with endothelial dysfunction and persistent inflammation, may partly explain this association [10,11]. However, this finding should be interpreted cautiously because renal dysfunction itself can increase circulating homocysteine concentrations through impaired metabolism and clearance. Consequently, the higher tHcy concentrations observed in participants with nephropathy may reflect renal involvement, poorer metabolic status, an increased cardiovascular risk-factor burden, or a combination of these mechanisms, rather than an independent vascular effect of homocysteine. Residual confounding related to renal function, nutritional status, age, medication use, and genetic determinants of homocysteine metabolism cannot be excluded. Experimental evidence also suggests that elevated homocysteine may further contribute to renal and vascular injury [10,11]; however, the cross-sectional nature of the present study does not allow the direction of these relationships to be established. These considerations reinforce the need to interpret plasma homocysteine within the broader clinical and metabolic context of T1DM rather than as an isolated marker of cardiovascular risk.

Although the associations between plasma homocysteine and lipid parameters were less pronounced than those observed for HbA1c and diabetes duration, positive correlations with LDL cholesterol and triglyceride concentrations were identified. These findings suggest that elevated homocysteine should not be considered an isolated cardiovascular risk factor but rather part of a broader network of metabolic abnormalities contributing to vascular injury in T1DM. Experimental studies have shown that excess homocysteine promotes oxidative modification of low-density lipoproteins, impairs endothelial integrity, enhances vascular smooth muscle cell proliferation, and increases platelet activation, thereby amplifying the atherogenic effects of dyslipidemia [10,11,19-21]. Consequently, the coexistence of hyperhomocysteinemia with conventional cardiovascular risk factors may accelerate the progression of endothelial dysfunction from an early age.

An important contribution of the present study is the evaluation of the discriminative performance of plasma homocysteine using receiver operating characteristic analysis. The observed AUC of 0.82 indicates good discriminative ability for identifying participants classified as having moderate-to-high cardiovascular risk according to the study-specific composite cardiovascular risk assessment, which was constructed independently of plasma tHcy. Although plasma homocysteine is unlikely to replace established clinical indicators such as HbA1c, diabetes duration, or renal assessment, our findings suggest that it may provide complementary information regarding cardiovascular risk-factor burden. These findings support further investigation of plasma homocysteine within multimarker approaches to cardiovascular risk assessment, particularly in pediatric patients presenting multiple established cardiovascular risk factors.

Current international recommendations, including the ISPAD Clinical Practice Consensus Guidelines and the American Diabetes Association Standards of Care, emphasize optimization of glycemic control together with systematic evaluation of conventional cardiovascular risk factors, including hypertension, dyslipidemia, obesity, renal involvement, and healthy lifestyle behaviors [1,3,4]. At present, routine measurement of plasma homocysteine is not recommended because prospective evidence demonstrating additional clinical benefit remains insufficient. Nevertheless, the results of our study, together with those of Rawshani et al. and other contemporary investigations, suggest that homocysteine may offer additional prognostic value in selected pediatric patients with poor metabolic control, prolonged disease duration, or established microvascular complications [5,6,13-15,18]. Larger prospective studies will be necessary to determine whether the incorporation of plasma homocysteine into pediatric cardiovascular risk algorithms improves early identification of patients who may benefit from intensified preventive strategies.

This study has several strengths that should be highlighted. To our knowledge, it represents one of the first investigations from North Africa specifically evaluating plasma homocysteine as a potential biomarker of cardiovascular risk in children and adolescents with T1DM. In addition to assessing homocysteine concentrations, we performed a comprehensive evaluation of demographic, clinical, metabolic, renal, and cardiovascular variables, allowing a broader characterization of cardiovascular risk in this population. The use of multivariable logistic regression and ROC curve analysis further strengthened the interpretation of our findings by identifying independent predictors of hyperhomocysteinemia and estimating its ability to discriminate patients with increased cardiovascular risk.

Nevertheless, several limitations should be acknowledged when interpreting these results. First, the cross-sectional design precludes establishing temporal or causal relationships between plasma homocysteine and cardiovascular risk factors. Second, the relatively small sample size and the single-center recruitment may limit the generalizability of our findings to other pediatric populations. Furthermore, the limited number of participants with hyperhomocysteinemia may have reduced the stability and precision of the multivariable regression estimates; therefore, these findings should be considered exploratory and require confirmation in larger cohorts. In addition, several determinants of homocysteine metabolism, including serum vitamin B12, folate and vitamin B6 concentrations, estimated glomerular filtration rate, dietary habits, and genetic polymorphisms involved in one-carbon metabolism, were not systematically assessed. Additionally, hyperhomocysteinemia was defined using a single laboratory-derived cutoff (>15 µmol/L) rather than age- and sex-specific pediatric reference intervals. As tHcy concentrations vary with age and sex during childhood and adolescence, particularly during puberty [30], this approach may have resulted in a conservative classification of elevated tHcy, especially in younger participants. Finally, direct imaging markers of subclinical vascular injury, such as carotid intima-media thickness, pulse wave velocity, or endothelial function testing, were not available and therefore could not be correlated with plasma homocysteine concentrations.

Despite these limitations, the present findings provide additional evidence supporting an association between elevated plasma homocysteine, poor metabolic control, longer diabetes duration, diabetic nephropathy, and an unfavorable cardiovascular risk-factor profile in pediatric T1DM [1,3,4,15,18-21]. Plasma homocysteine should therefore be considered an investigational adjunctive marker rather than an established predictive or prognostic biomarker. Future multicenter prospective studies including larger cohorts, standardized homocysteine measurements, appropriate adjustment for renal function and vitamin status, and direct measures of vascular structure and function are needed to validate these findings and determine whether plasma homocysteine provides clinically meaningful incremental information beyond established cardiovascular risk factors.

## Conclusions

The present study supports the potential role of plasma homocysteine as an adjunctive biomarker for cardiovascular risk assessment in children and adolescents with T1DM. Hyperhomocysteinemia was associated with poor glycemic control, longer diabetes duration, diabetic nephropathy, and a greater burden of established cardiovascular risk factors. Plasma tHcy also demonstrated good discriminative ability for distinguishing participants classified as having moderate-to-high cardiovascular risk according to the study-specific composite cardiovascular risk assessment. These findings suggest that plasma homocysteine may provide complementary information for cardiovascular risk assessment in pediatric T1DM.

Although glycated hemoglobin remains a cornerstone of metabolic monitoring, plasma homocysteine may provide additional information regarding cumulative metabolic and cardiovascular risk-factor burden. Given the study’s single-center, cross-sectional design and relatively small sample size, these findings should be considered exploratory. Larger prospective multicenter studies are needed to validate the observed associations and determine whether plasma homocysteine provides clinically meaningful incremental value beyond established cardiovascular risk factors in children and adolescents with T1DM.
